# Resistance of SARS-CoV-2 Delta variant to neutralization by BNT162b2-elicited antibodies in Asians

**DOI:** 10.1016/j.lanwpc.2021.100276

**Published:** 2021-09-20

**Authors:** Bei Wang, Yun Shan Goh, Siew-Wai Fong, Barnaby Edward Young, Eve Zi Xian Ngoh, Jean-Marc Chavatte, Siti Nazihah Mohd Salleh, Nicholas Kim-Wah Yeo, Siti Naqiah Amrun, Pei Xiang Hor, Chiew Yee Loh, Chia Yin Lee, Yi-Hao Chan, Zi Wei Chang, Matthew Zirui Tay, Angeline Rouers, Anthony Torres-Ruesta, Guillaume Carissimo, Mun Kuen Soh, Raphael Tze Chuen Lee, Yani Xu, Surinder Pada, Raymond Tzer Pin Lin, Yee-Sin Leo, David C. Lye, Sebastian Maurer-Stroh, Lisa F.P. Ng, Laurent Renia, Cheng-I Wang

**Affiliations:** 1 Singapore Immunology Network, A*STAR, Singapore; 2 A*STAR Infectious Diseases Labs, A*STAR, Singapore; 3 National Centre for Infectious Diseases, Singapore; 4 Department of Infectious Diseases, Tan Tock Seng Hospital, Singapore; 5 Lee Kong Chian School of Medicine, Nanyang Technological University, Singapore; 6 Bioinformatics Institute, A*STAR, Singapore; 7 Division of Infectious Diseases, Ng Teng Fong General Hospital, Singapore; 8 Yong Loo Lin School of Medicine, National University of Singapore and National University Health System, Singapore; 9 Department of Biological Sciences, National University of Singapore, Singapore; 10 Department of Biochemistry, National University of Singapore, Singapore; 11 Institute of Infection, Veterinary and Ecological Sciences, University of Liverpool, Liverpool, UK; 12 NIHR Health Protection Research Unit in Emerging and Zoonotic Infections, Liverpool, UK; 13 School of Biological Sciences, Nanyang Technological University, Singapore

SARS-CoV-2 was first detected in late December 2019, however, in the few months that followed, the resultant COVID-19 disease has developed into a devastating pandemic around the world [1]. This has led to a race to produce a safe and efficacious vaccine in record time. In less than a year, BNT162b2, an mRNA-based vaccine developed by BioNTech-Pfizer [2], became the first ever approved COVID-19 vaccine in December 2020. However, this coincided with the emergence of multiple variants. These variants, though originated from different countries such as United Kingdom, South Africa, Brazil, India and United States of America in five different continents, have now spread globally, resulting in second and third waves of COVID-19 cases around the world. In order to prioritize the global monitoring and research to guide the ongoing response to the pandemic, the World Health Organization (WHO), in partner with various authorities worldwide, prompted the characterization of specific Variants of Interest (VOIs) and Variants of Concern (VOCs) [3]. As of 15^th^ June 2021, four VOCs have been defined as they are associated with one or more of the following features: increase in transmissibility, increase in virulence, and decrease in effectiveness of available diagnostics, vaccines or therapeutics. These include the Alpha (B.1.1.7), Beta (B.1.351), Gamma (P.1) and Delta (B.1.617.2) variants. In addition to these four VOCs, another seven strains were classified as VOIs, including Epsilon (B.1.427/B.1.429), Zeta (P.2), Eta (B.1.525), Theta (P.3), Iota (B.1.526), Kappa (B.1.617.1) and Lambda (C.37) [3].

Since the FDA issued the emergency use authorization (EUA) of the COVID-19 vaccines in late 2020, more than 2.89 billion vaccine shots have been administered globally as of June 27 2021 [4], among which a significant portion of the total shots came from the BNT162b2, an mRNA-based vaccine developed by BioNTech-Pfizer. However, the rapid evolution of SARS-CoV-2 variants has raised serious concerns in viral escape from vaccine-elicited immunity. Even in countries with an active mRNA-based COVID-19 vaccine program, there is a surge in the number of COVID-19 cases associated with the Delta variant (B.1.617.2) [1,3]. Immune sera, collected from individuals vaccinated with the BNT162b2 vaccine in USA, have strong neutralizing capacitiy against SARS-CoV-2 WA-1/2020 reference strain and the Alpha variant (B.1.1.7), and moderate neutralizing capacitiy against the Beta (B.1.351) strain [5]. Though it was still able to confer protection against symptomatic infection, vaccination with BNT162b2 in Europe and in the USA seems to have induced less potent neutralizing antibody responses against the newly emerged Delta variant B.1.617.2 [6].

Since receiving the first batch of COVID-19 vaccines in December 2020, Singapore has kick-started its mass vaccination drive. However, following the first detection of the Delta variant in March 2021, the number of cases has increased sharply and soon it became the dominant local strain (45.7%), which accounted for the recent third wave of COVID-19 sporadic outbreak (Figure 1) [7]. Among this third wave of COVID-19 cases was a number of vaccine breakthrough cases [7]. It remains to be determined whether Asian populations vaccinated with the BNT162b2 vaccine might remain vulnerable to the COVID-19 disease when confronted with the variants, such as the Delta variant. To answer this question, we first screened a set of plasma samples obtained from 50 study participants (22-69 years of age) of various Asian ethnicities (Supplementary Table 1), collected prior to vaccination (Before) and at the median 71 days after the second immunization with BNT162b2 (After), for their specific IgG against the full length WT SARS-CoV-2 S protein expressed on the surface of the HEK293T cells, using a S protein flow-based assay (SFB assay) [8]. In addition, the plasma samples were also subjected to evaluation for their specific anti-Nucleocapsid IgG levels by Elecsys® Anti-SARS-CoV-2 N immunoassay. All study subjects were SARS-CoV-2 seronegative before vaccination. Two doses of BNT162b2 vaccination elicited robust anti-Spike IgG in all individuals (Supplementary Figure 1A), while the anti-Necleocapsid IgG levels were not detectable across all samples either before or after vaccination (Supplementary Figure 1B), indicating that all study subjects had not encountered any prior SARS-CoV-2 infection and the anti-Spike responses were solely elicited by the BNT162b2 vaccination. The immune plasma samples were also tested against previously identified immunodominant B-cell linear epitopes S14P5, S20P2 and S21P2 outside the RBD on the spike glycoprotein [9]. IgG levels against these three epitopes increased after two doses of vaccination (Supplementary Figure 1C), confirming recent study that reported an induction of antibodies against diverse regions of S protein following SARS-CoV-2 spike mRNA vaccination [10].

**Figure 1:**
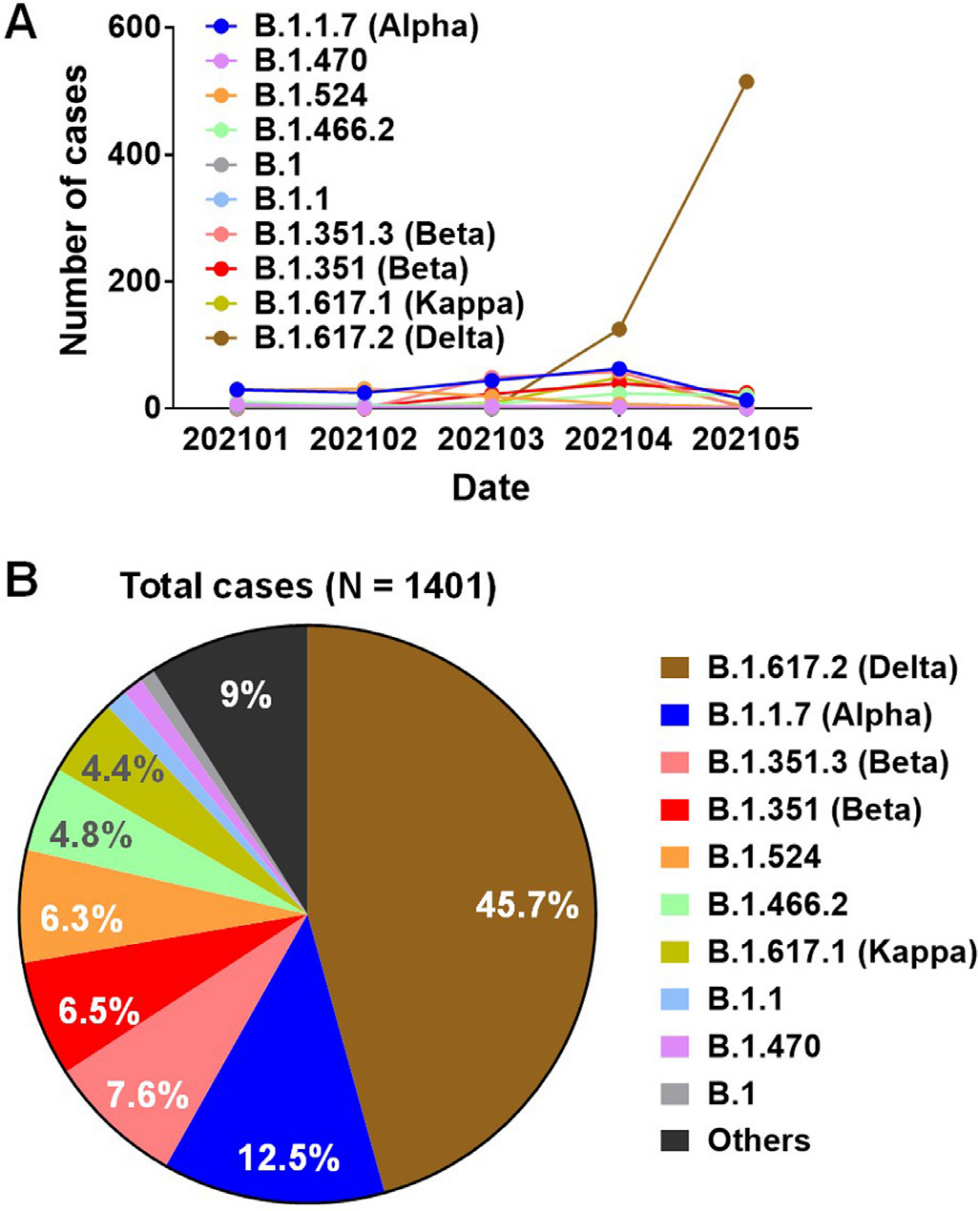
**SARS-CoV-2 lineages prevalent in Singapore in January to May 2021.** The number of cases infected by various SARS-CoV-2 variants collected from January to May 2021 in Singapore with genomes sequenced and shared via GISAID are shown in both **(A)** timeline presentation and (**B**) Pie chart with percentages of each variant indicated

Following the confirmation of the development of a robust vaccine-induced antibody response, we then generated the lentivirus-based SARS-CoV-2 pseudoviruses bearing the Spike proteins of the Delta variant and examined the neutralizing capability of the vaccine-induced antibody response against the reference Wuhan strain (WT), the other VOCs (Alpha, Beta, Gamma), and another two relevant VOIs – Kappa and Epsilon that contained the same L452R mutation at the receptor binding domain (RBD) as the Delta variant (Figure 2A) [3]. Using CHO cells over-expressing human ACE2 protein (CHO-ACE2), we found that the pseudovirus of all tested variants infected the cells more efficiently than the Wuhan reference strain (WT) (*p<0.0001) (Supplementary Figure 2). This may explain the clinical observations of the enhanced infectivity and transmission of these SARS-CoV-2 variants in human population. Intriguingly, Wang et al. have showed similar luciferase RLU values of B.1.1.7 and B.1.351 pseudovirus strains in relative to the D614G reference strain [11], which is different from our experimental setting using the ancestral Wuhan strain as the benchmark, suggesting that the enhanced infectivity observed in our study was largely attributed to the D614G mutation across all tested variants, which had been confirmed to be associated with conformational changes of Spike trimer and increased infectivity [12]. The neutralization potency against different variant strains can be clearly differentiated when the plasma samples were applied at the 1:20 dilution. Overall, the antibody neutralization activities against B.1.1.7, P.1 or B.1.429 strains were somewhat reduced compared to the WT strain, even though the reduction is statistically insignificant (Figure 2B). This finding slightly differs from some recent reports showing significant drop in antibody neutralization efficacies against these strains [5,13,14]. We hypothesized that the genetic variations among different populations which can develop different antibody repertoires, may also contribute to the fine specificity of antibody response observed in the current study. Corroborating with the recent report [5], there was a significant drop in antibody response against B.1.351 strain (average 57.1% neutralization compared to an average 76% neutralization against WT strain). The most prominent decline was detected in the neutralization potency against the Delta variant B.1.617.2 (average 37.4% neutralization) but not the Kappa variant B.1.617.1 (average 58% neutralization) (Figure 2B). As high as 74% of the plasma samples (37 out 50) can be classified as either ‘Weak neutralizers’ or ‘Non-neutralizers” against B.1.617.2 due to their inability to neutralize more than 50% of pseudovirus particles at the 20-fold plasma dilution (Figure 2C). This observation provided a plausible explanation for the recent surge in the cases associated with B.1.617.2 in Singapore (Figure 1). Interestingly, when we applied correlation analysis of percentages of neutralization among 50 plasma samples between WT strain and each of the 6 variant strains, we have observed significant levels of correlation of the data points across all 6 variants (Supplementary Figure 3). This proportional changes in antibody neutralization efficiency of variant strains were also reported by another recent study in comparing the B.1.617.1 Kappa variant and the reference Victoria strain [15]. A further analysis on the effects of gender, age and ethnicity did not callout any significant correlations with any of these factors for all 7 tested strains (Supplementary Figure 4). Previous studies have reported that male COVID-19 patients are more at risk for worse outcomes and high fatality than females, suggesting differential immune responses between two sexes to SARS-CoV-2 infection [16]. However, we did not observe any gender differences in antibody responses in our study. This is likely due to the limitation of this study as most of study subjects included in the current study are females (82%, Supplementary Table 1) and hence there is an imbalanced gender distribution. In addition, the discrepancy between our finding and another recent report which showed that increased age significantly correlated with reduced neutralizing antibody titers [6] may be due to the small sample size in the current study.

**Figure 2:**
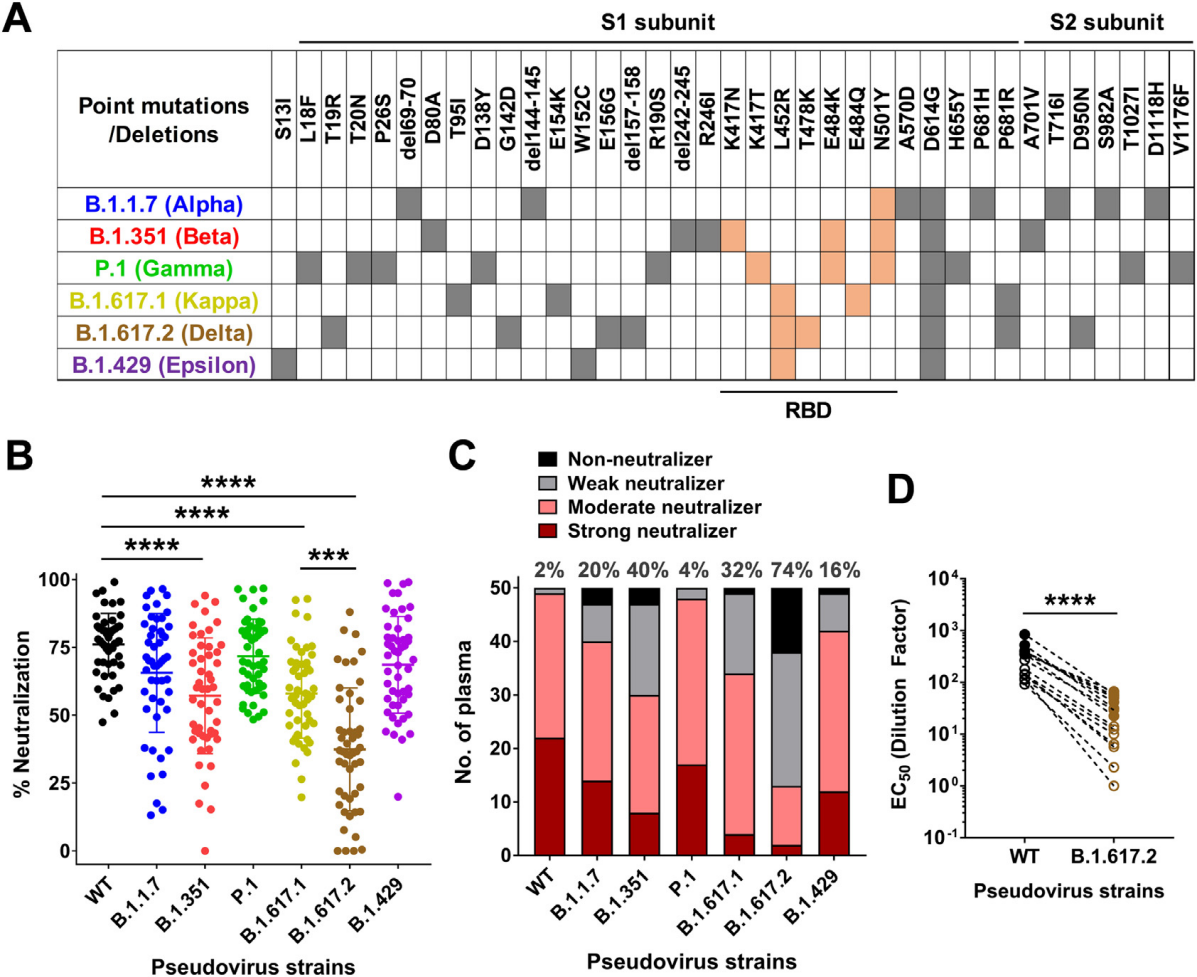
**Pseudovirus neutralization capacity of 50 plasma samples from BNT162b2 (Pfizer-BioNTech) vaccinees against SARS-CoV-2-Spike pseudoviruses bearing the Wuhan reference strain or the other 6 variant strains.** (**A**) Amino acid changes (point mutations or deletions) relevant to the Wuhan reference strain of different SARS-CoV-2 variants, including B.1.1.7 (Alpha), B.1.351 (Beta), P.1 (Gamma), B.1.617.1 (Kappa), B.1.617.2 (Delta), and B.1.429 (Epsilon). Orange filled boxes: changes at the receptor binding domain (RBD); Grey filled boxes: changes outside the RBD. (**B**) 50 plasma samples from BNT162b2 vaccinees collected 68 to 77 days (median 71 days) after the second dose of vaccination were tested at 1:20 dilutions for their neutralization potency against Wuhan reference strain (WT) or the other 6 variant strains. Statistical analysis was carried out to compare any two different pseudoviruses using Kruskal-Wallis tests followed by post hoc Dunn's multiple comparisons tests (*** *P* ≤ 0.001, **** *P* ≤ 0.0001). Only the comparison between the Wuhan reference strain (WT) and each of the other 6 variant strains plus that between the B.1.617.1 strain and the B.1.617.2 strain were shown in the figure. **(C)** Each individual plasma sample was arbitrarily defined as either ‘Strong neutralizer (red bar)’, or ‘Moderate neutralizer (pink bar)’, or ‘Weak neutralizer (grey bar)’ or ‘Non-neutralizer (black bar)’ if the percentage of neutralization at the 1:20 dilution is higher than 80%, between 50% to 80%, between 20 to 50%, and lower than 20%, respectively. The numbers indicated the combined percentage of ‘Weak neutralizers’ and ‘Non-neutralizers’ (0-50% neutralization) out of total 50 plasma samples for each different pseudovirus strains. (**D**) Comparison of EC50 values of neutralization between WT (black circles), and the B.1.617.2 strain (brown circles) pseudoviruses from 15 selected donor samples of either high responders against the B.1.617.2 strain (filled circles, n = 7) or low responders against the B.1.617.2 strain (empty circles, n = 8). Statistical analysis was carried out using paired two-tailed t-test ( **** *P* ≤ 0.0001).

To further quantify the differences in neutralizing potency against B.1.617.2 strain compared to the ancestral strain, we selected 7 high responders (70-88% neutralization against B.1.617.2 pseudovirus at 1:20 dilution) and 8 low responders (0-32% neutralization against B.1.617.2 pseudovirus at 1:20 dilution) on a full dose neutralization curve analysis (Supplementary Figure 5). The results showed an average 9.9-fold drop in EC_50_ in high responders (5.1 to 16.7-fold) and 14.7-fold drop in low responders (9.2 to 113.9-fold), which combined to confer an average 10.9-fold drop in the 15 selected study participants (Figure 2D).

Lastly, we performed correlation analyses to compare a series of self-developed and commercial assays on the anti-Spike antibody responses (binding or receptor blockade) with the pseudovirus neutralization assay against various SARS-CoV-2 variants. Interestingly, we could not detect any correlation between pseudovirus neutralization potency and the receptor blocking efficiency measured by the GenScript cPass^TM^ SARS-CoV-2 Neutralization Antibody Detection Kit [17] for all 7 SARS-CoV-2 variants tested in this study, except a weak correlation can be observed for WT strain (*p* = 0.028) (Supplementary Figure 6). This suggests that an immunoassay using solely the RBD of the wildtype SARS-CoV-2 Spike protein, even it is designed as a functional biochemical method based on RBD/ACE2 receptor blocking, does not represent or accurately predict the neutralization potency against the SARS-CoV-2 variants in the real world. This is likely because all of these emerging SARS-CoV-2 variants also mutated at regions outside the RBD such as the N-terminal domain (NTD) and S2 domain, some of which may attenuate or even abolish the neutralizing ability of the antibodies recognizing the epitopes at or near the site of the mutations [10]. On the contrary, a moderately positive correlation can be detected when we compared pseudovirus neutralization data with assays measuring antibody binding to full-length WT Spike proteins including our in-house SFB assay [8] (Pearson's *r* = 0.425, *p* = 0.002) and the commercial Elecsys® Anti-SARS-CoV-2 S immunoassay developed by Roche Diagnostics (Pearson's *r* = 0.649, *p* < 0.0001). We also observed significant correlation with 3 variants using SFB assay and across all 6 variants using Elecsys® Anti-SARS-CoV-2 S immunoassay (Supplementary Figure 7), suggesting that the antibody binding assays involving the full-length Spike protein, even though they are limited to the WT Spike sequence, may provide a qualitative assessment of the level of neutralizing antibodies as well as a quick and sound prediction on the neutralizing capacity against various SARS-CoV-2 variants. More interestingly, IgG response against the potential immunodominant epitope S14P5 [9], also showed significant positive correlation with pseudovirus neutralization activity against 2 variants including the Alpha variant B.1.1.7 (Supplementary Figure 8), which contains a single point mutation A570D in the region where the neutralizing peptide S14P5 is localized. However, this point mutation does not appear to compromise the performance of S14P5 epitope in estimating neutralization efficiency against the B.1.1.7 variant.

Collectively, our results showed a prominent decline in neutralizing antibody levels against the B.1.617.2 Delta variant, in line with the breakthrough infection in BNT162b2 vaccinated health care workers in Singapore (https://www.moh.gov.sg/news-highlights/details/ministerial-statement-11May). It also suggested that the current vaccines may not be able to induce sufficient protection against the Delta variant among the current VOCs, highlighting an urgent need for further booster immunizations to the populations that exhibited lower vaccine-induced immune responses against emerging variants as well as developing next generation vaccines of broad-spectrum neutralization capacity. In addition, our comprehensive comparative analyses on anti-Spike antibody responses between different assays suggest a scenario of using full-length Spike protein-based antibody binding assays as a fast screening step for the initial assessment of vaccine-elicited protection against both WT SARS-CoV-2 strain and the emerging variants, although neutralization assays using either pseudoviruses or live viruses remain as the gold standard for monitoring levels of humoral immunity.

BW, YSG, SWF, BEY, and EZXN contributed equally. LFPN, LR, and CIW are joint senior authors. Funding details, acknowledgments, declaration of competing interests can be found in the appendix. All data and all figures and statistical analysis presented in this Correspondence are also included in the appendix.

## Declaration of Competing Interest

A patent application for the SFB assay has been filed (Singapore patent 10202009679P: A Method Of Detecting Antibodies And Related Products. YSG, LFPN, and LR). A patent application on the identified linear epitopes S14P5 and S20P2 has also been filed (PCT/SG2021/050178: Antibody-binding linear B cell epitopes of SARS-CoV and SARS-CoV-2. NKWY, SNA, GC, and LFPN). All other authors declare no conflict of interest.

## Appendix Supplementary materials

Image, application 1
