# Supplementary material for: Resistance of SARS-CoV-2 Delta variant to neutralization by BNT162b2-elicited antibodies in Asians
Source: Lancet Reg Health West Pac. 2021 Sep 20;15:100276. doi: 10.1016/j.lanwpc.2021.100276 (PMC8450274; doi:10.1016/j.lanwpc.2021.100276)
Supplement: Supplementary file 1 [file mmc1.docx]

**Appendix for**

**Resistance of SARS-CoV-2 Delta variant to neutralization by BNT162b2-elicited antibodies in Asians**

Bei Wang^1^†, Yun Shan Goh^2^†, Siew-Wai Fong^2^†, Barnaby Edward Young^3,4,5^†, Eve Zi Xian Ngoh^1^†, Jean-Marc Chavatte^3^, Siti Nazihah Mohd Salleh^1^, Nicholas Kim-Wah Yeo^2^, Siti Naqiah Amrun^2^, Pei Xiang Hor^2^, Chiew Yee Loh^2^, Chia Yin Lee^1^, Yi-Hao Chan^2^, Zi Wei Chang^2^, Matthew Zirui Tay^2^, Angeline Rouers^2^, Anthony Torres-Ruesta^2^, Guillaume Carissimo^2^, Mun Kuen Soh^1^, Raphael Tze Chuen Lee^6^, Yani Xu^6^, Surinder Pada^7^, Raymond Tzer Pin Lin^3,8^, Yee-Sin Leo^3,4,5,8^, David C. Lye^3,4,5,8^, Sebastian Maurer-Stroh^2,6,9^, Lisa F.P. Ng^2,10,11,12^*, Laurent Renia^2,5,13^*, Cheng-I Wang^1^*

**Affiliations**

^1^Singapore Immunology Network, Agency for Science, Technology and Research (A*STAR), Immunos, Biopolis, Singapore 138648, Singapore

^2^A*STAR Infectious Diseases Labs (A*STAR ID Labs), Agency for Science, Technology and Research (A*STAR), Immunos, Biopolis, Singapore 138648, Singapore

^3^National Centre for Infectious Diseases, 16 Jalan Tan Tock Seng, Singapore 308442, Singapore

^4^Department of Infectious Diseases, Tan Tock Seng Hospital, 11 Jalan Tan Tock Seng, Singapore 308433, Singapore

^5^Lee Kong Chian School of Medicine, Nanyang Technological University, 11 Mandalay Road, Singapore 308232, Singapore

^6^Bioinformatics Institute, Agency for Science Technology and Research (A*STAR), Singapore

^7^Division of Infectious Diseases, Ng Teng Fong General Hospital, 1 Jurong East Street 21, Singapore 609606, Singapore.

^8^Yong Loo Lin School of Medicine, National University of Singapore and National University Health System, 10 Medical Drive, Singapore 117597, Singapore

^9^Department of Biological Sciences, National University of Singapore, Singapore

^10^Department of Biochemistry, Yong Loo Lin School of Medicine, National University of Singapore, Singapore

^11^Institute of Infection, Veterinary and Ecological Sciences, University of Liverpool, Liverpool, UK.

^12^NIHR Health Protection Research Unit in Emerging and Zoonotic Infections, Liverpool, UK.

^13^School of Biological Sciences, Nanyang Technological University, 60 Nanyang Drive, Singapore 637551, Singapore

†These authors contributed equally to this work.

*Correspondence to: Wang Cheng-I [wang_chengi@immunol.a-star.edu.sg](mailto:wang_chengi@immunol.a-star.edu.sg), Laurent Renia [renia_laurent@IDLabs.a-star.edu.sg](mailto:renia_laurent@IDLabs.a-star.edu.sg), and Lisa F.P. Ng [lisa_ng@IDLabs.a-star.edu.sg](mailto:lisa_ng@IDLabs.a-star.edu.sg)

**Contents**

Supplementary Table 1

Supplementary Figures 1-8

Supplementary Methods

Supplementary References

**Supplementary Table 1. Demographics of BNT162b2 vaccinees in this study.**

| Demographics (N = 50) Count (%) | |
| --- | --- |
| Age |  |
| 22 – 39 years | 32 (64%) |
| 40 – 69 years | 18 (36%) |
| Gender | |
| Male | 9 (18%) |
| Female | 41 (82%) |
| Ethnicity | |
| Chinese | 33 (66%) |
| Malay | 5 (10%) |
| Indian | 4 (8%) |
| Others (Filipino, Vietnamese) | 8 (16%) |


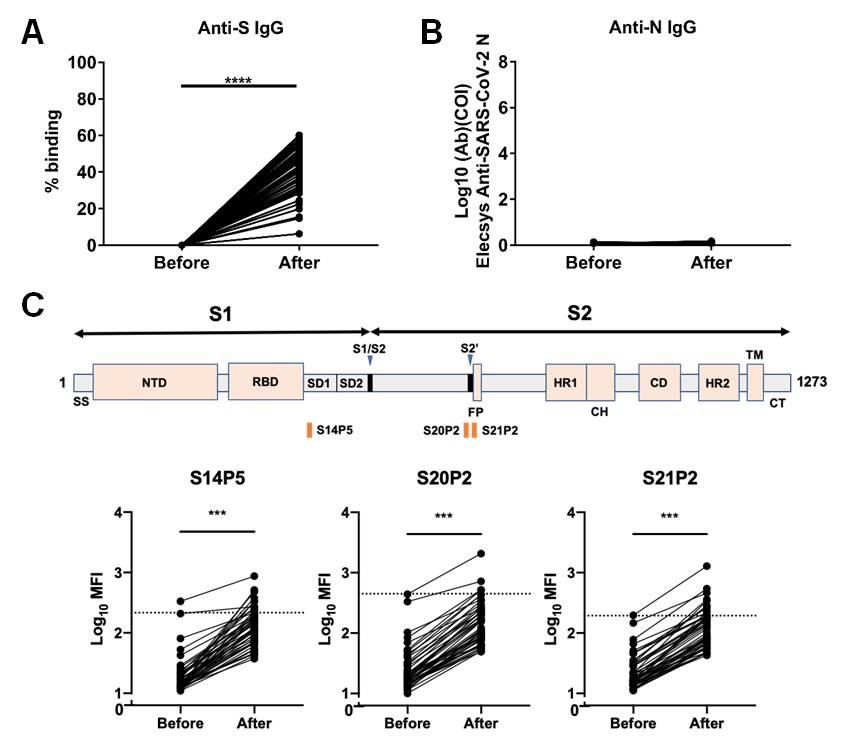


**Supplementary Figure 1. SARS-CoV-2 spike protein specific antibody responses to the BNT162b2 mRNA COVID-19 vaccine. (A)** Antibody binding to full length WT S protein. Plasma samples from BNT162b2 vaccinees (n=50) were collected prior to vaccination (Before) and at 68 to 77 days (After; median 71 days) after the second dose of vaccination were screened at 1:100 dilution for specific IgG against WT full length original Wuhan SARS-CoV-2 S protein expressed on the surface of HEK293T cells. Data are shown as mean ± SD of two independent experiments. Statistical analysis was carried out on the paired samples using Friedman test, followed by post hoc Dunn’s multiple comparison tests. *P* values for comparisons between the groups are shown, where **** indicates *p* ≤ 0.0001. **(B)** The 50 plasma samples were also screened for anti-nucleocapsid antibodies using the Roche’s Elecsys Anti-SARS-CoV-2 N test. COI, Cutoff index. **(C)** Schematic diagram showing the localization of SARS-CoV-2 specific IgG epitopes S14P5, S20P2 and S21P2 on primary structure of the Spike protein. S14P5 is shown to localize in close proximity to the receptor binding domain (RBD), whereas S21P2 cover part of the fusion peptide. Domains of the Spike protein are labelled with coloured box. SS, signal sequence; NTD: N-terminal domain; RBD: receptor-binding domain; SD1: Subdomain 1; SD2: Subdomain 2; S1/S2: S1/S2 protease cleavage site; S2′: S2′ protease cleavage site; FP: fusion peptide; HR1: heptad repeat 1; CH: central helix; CD: connector domain; HR2: heptad repeat 2; TM: transmembrane domain; CT: cytoplasmic tail. Anti-peptide IgG responses (1:1000 plasma dilution) were measured in 50 vaccinees prior to vaccination (Before) and at 68 to 77 days (After; median 71 days) after the second dose of vaccination. Dotted line indicates mean + 3SD of healthy donors (n=28). Statistical analysis was carried out on the paired samples using Wilcoxon Matched-Pairs Signed Ranks Test, where *** indicates *p* ≤ 0.001.

**Supplementary Figure 2. Entry efficiency of different pseudovirus strains.** The infectivity of different pseudovirus strains bearing the Spike proteins of either the Wuhan reference strain (WT) or the other 6 variants as shown by raw Relative Luminescence Unit (RLU) values. Statistical analysis was carried out to compare any two different pseudoviruses using ordinary one-way ANOVA tests followed by Turkey’s multiple comparisons tests (**** *p* ≤ 0.0001). Only the comparison between the Wuhan reference strain (WT) and each of the other 6 variant strains were shown in the figure.


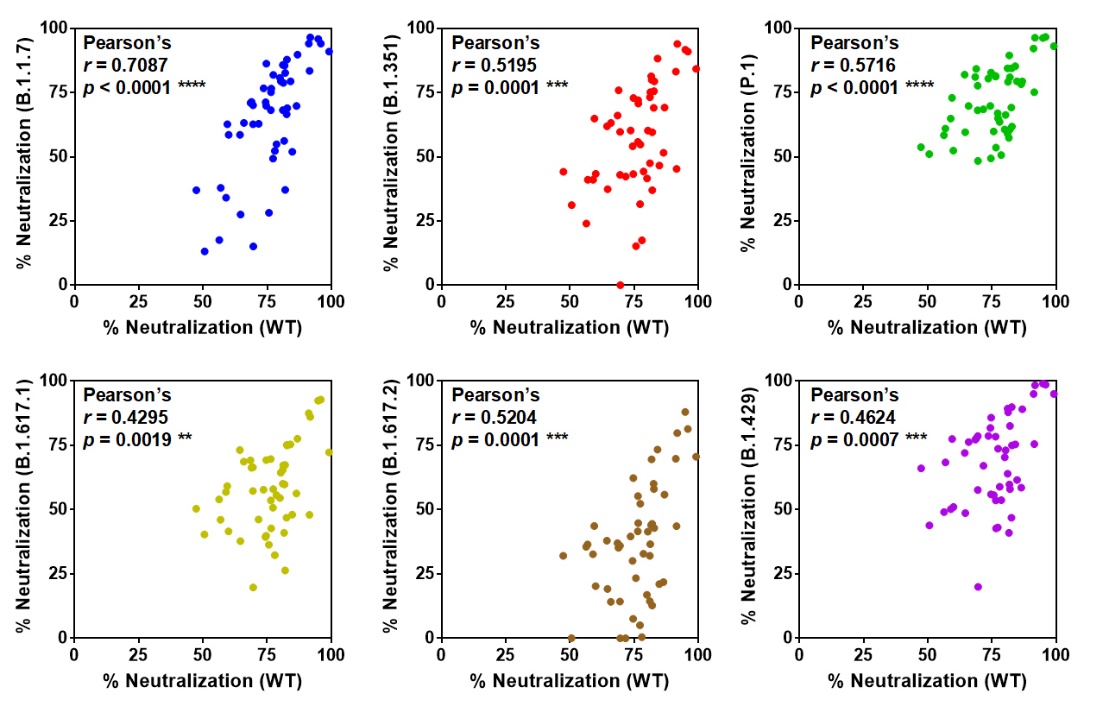


**Supplementary Figure 3. Correlation analysis of antibody neutralization potency against WT pseudovirus and each of the variant pseudoviruses.** The correlation analysis was performed to compare percent neutralization among 50 BNT162b2 vaccinees at 68 to 77 days (median 71 days) after the second dose of vaccination against each of the 6 different variant pseudovirus strains tested at 1:20 dilution and that against the WT pseudovirus. Pearson’s correlation coefficient (Pearson’s *r*) and *p* values are shown, where ** indicates *p* ≤ 0.01, *** indicates *p* ≤ 0.001, and **** indicates *p* ≤ 0.0001.

**Supplementary Figure 4. Effect of gender, age and ethnicity on antibody neutralization against different pseudovirus strains.** Dot plots of percent neutralization against Wuhan reference strain (WT, black), the B.1.1.7 strain (blue), the B.1.351 strain (red), the P.1 strain (green), the B.1.617.1 strain (lime green), the B.1.617.2 strain (brown) and the B.1.429 strain (purple) from 50 plasma samples (at 1:20 dilution) of BNT162b2 vaccinees at 68 to 77 days (median 71 days) after the second dose of vaccination, presented in groups of (**A**) different genders of males (n=9, triangles) and females (n=41, circles), or (**B**) different ages of younger than 40 (n=32, circles) and equal or older than 40 (n=18, triangles), or (**C**) different ethnicities of Chinese (n=33, circles), Malay (n=5, triangles), Indian (n=4, squares) and Others (diamonds, n=8, 7 Filipinos and 1 Vietnamese). Statistical analysis was carried out to compare vaccinees of different genders in (**A**) and ages in (**B**) using two-tailed t-test. Statistical analysis was carried out to compare vaccinees of different ethnicity groups in (**C**) using Kruskal-Wallis tests followed by post hoc Dunn’s multiple comparisons tests. There is no significant difference between any different groups of vaccinees against all different pseudovirus strains.

**Supplementary Figure 5. Pseudovirus neutralization curves of 15 selected plasma samples from BNT162b2 vaccinees.** Plasma samples of selected high responders to BNT162b2 vaccines (n = 7) (**A**) or low responders (n = 8) (**B**) were incubated with pseudovirus expressing SARS-CoV-2 spike (S) glycoprotein 1 hour prior to infection of CHO-ACE2 cells for 48 hours. Infection levels were determined by luciferase assay, and percentage of neutralization is presented. Dose-response titration curves against Wuhan reference strain (WT, left panels) or the B.1.617.2 strain (right panels) at 1:10 to 1:6250 dilutions. Lines represent non-linear regression fit and data are shown as mean ± SD of duplicate wells. EC_50_ values were calculated using Quest Graph™ EC50 Calculator from AAT Bioquest, Inc (https://www.aatbio.com/tools/ec50-calculator) with top and bottom constraints set at 100% and 0% respectively.

**Supplementary Figure 6. Correlation analysis between antibody neutralization potency and receptor blocking activity.** Correlation analysis was performed to compare percent neutralization against each of the 7 different pseudovirus strains tested in this study measured by pseudovirus neutralization assay at 1:20 dilution and the percent inhibition of RBD/ACE2 interaction measured by cPass SARS-CoV-2 Neutralization Antibody Detection Kit (GenScript) in 50 vaccinees at 68 to 77 days (median 71 days) after second dose of vaccination. Pearson’s correlation coefficient (Pearson’s *r*) and *p* values are shown, where * indicates *p* ≤ 0.05.

**Supplementary Figure 7. Correlation analysis between antibody neutralization potency and Spike specific antibody binding.** (**A**) Correlation analysis was performed to compare percent neutralization against each of the 7 different pseudovirus strains tested in this study at 1:20 dilution and percent binding of S-specific IgG measured by SFB assay in 50 vaccinees at 68 to 77 days (median 71 days) after second dose of vaccination. (**B**) Correlation analysis was performed to compare percent neutralization against each of the 7 different pseudovirus strains tested in this study at 1:20 dilution and actual plasma concentration of S-specific antibodies (Log10) measured by Elecsys® Anti-SARS-CoV-2 S immunoassay (Roche Diagnostics) in 50 vaccinees at 68 to 77 days (median 71 days) after second dose of vaccination. Pearson’s correlation coefficient (Pearson’s *r*) and *p* values are shown, where * indicates *p* ≤ 0.05, ** indicates *p* ≤ 0.01, *** indicates *p* ≤ 0.001, **** indicates *p* ≤ 0.0001.


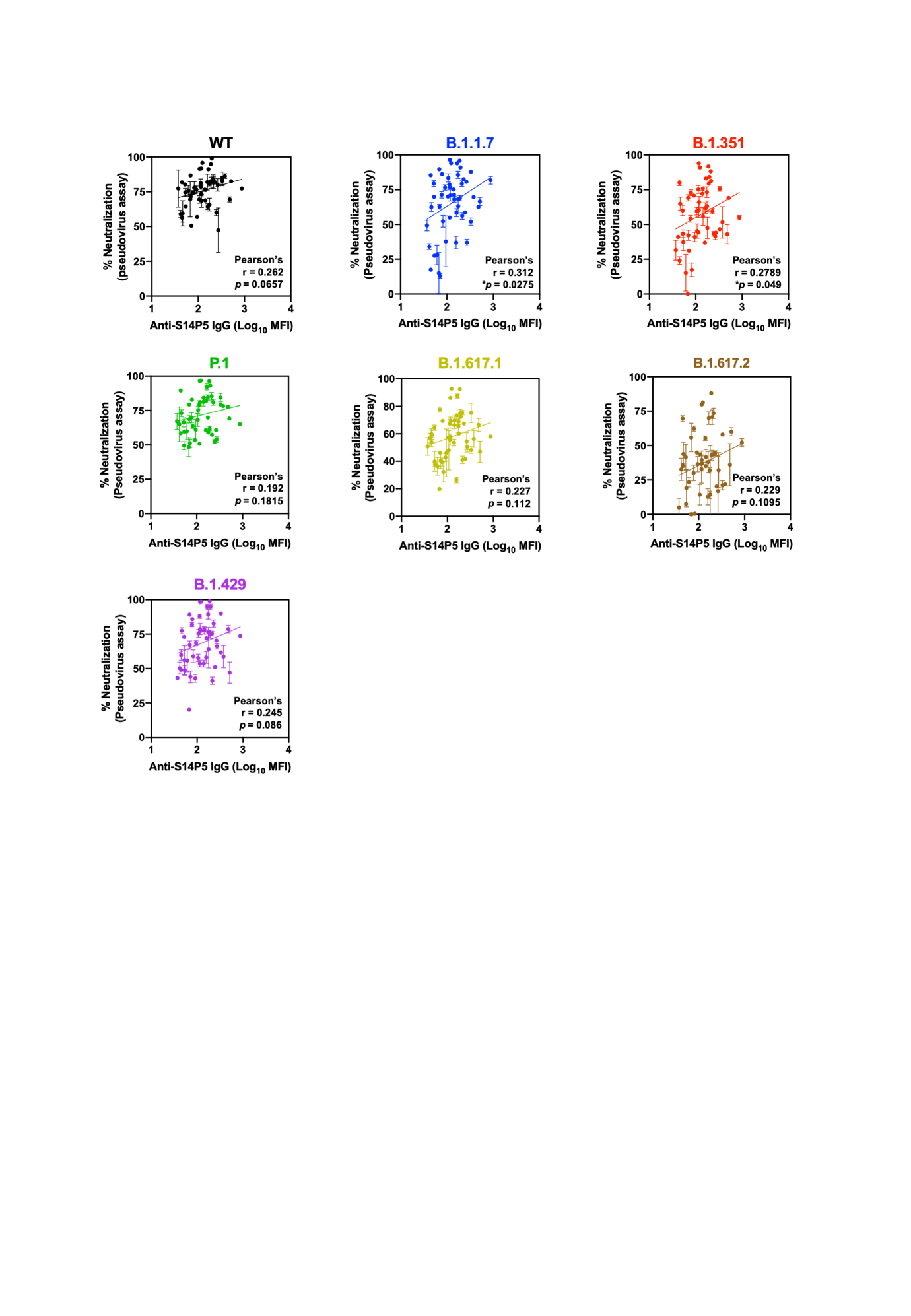


**Supplementary Figure 8. Correlation analysis between antibody neutralization potency and anti-S14P5 peptide IgG responses.** Correlation analysis was performed to compare percent neutralization against each of the 7 different pseudovirus strains tested in this study at 1:20 dilution and anti-S14P5 peptide IgG responses measured in Log_10_ Mean Fluorescent Intensity (MFI) in 50 vaccinees at 68 to 77 days (median 71 days) after second dose of vaccination. Pearson’s correlation coefficient (Pearson’s *r*) and *p* values are shown, where * indicates *p* ≤ 0.05.

Supplementary Methods

***Plasma samples for BNT162b2 vaccinees***

The study design and protocols for COVID-19 cohorts of vaccinees were approved by National Healthcare Group (NHG) Domain Specific Review Board (DSRB) and performed, following ethical guidelines in the approved studies IRB#2012/00917. Plasma samples were collected from a cohort of healthcare workers who received BNT162b2 vaccination at public hospitals in Singapore. Blood samples of 50 participants were collected on the day of vaccination and at 68 to 77 days (median 71 days) after second dose of vaccination.

***Generation of constructs expressing Spike genes of different variants***

The pTT5LnX-CoV-SP (expressing SARS-CoV-2 Spike protein, Genbank: YP_009724390.1, a kind gift from Dr. Brendon John Hanson, DSO National Laboratories) was used as a template plasmid to generate Spike genes of different variants using QuickChange Lightning Multi Site-Directed Mutagenesis Kit (Agilent, Cat#210513).

***S protein flow cytometry-based assay (SFB assay) for antibody detection***

The SFB assay was performed as previously described.^1^ Briefly, S protein-expressing cells were seeded at 1.5 x 10^5^ cells per well in 96 well V-bottom plates (Thermo Fisher Scientific, Cat#249570). After incubating the cells with diluted human plasma (1:100 in 10% FBS), the cells were incubated with a double stain, consisting of Alexa Fluor 647-conjugated anti-human IgG (Thermo Fisher Scientific, Cat#A21445; diluted 1:500) and propidium iodide (PI, Sigma-Aldrich, Cat#P4170; diluted 1:2500). Cells were read on BD Biosciences LSR4 laser and analyzed using FlowJo (Tree Star). Binding is determined by the percentage of GFP-positive S protein-expressing cells that are bound by specific antibody, indicated by the events that are Alexa Fluor 647- and FITC-positive. Quantification of binding of specific antibody binding to cells were analyzed following our previous study.

***Multiplex microbead-based immunoassay***

The multiplex microbead-based immunoassay was used to analyze IgG responses against three linear epitopes S14P5, S20P2, S21P2. Patient plasma samples were inactivated with Triton™ X-100 (Thermo Fisher Scientific) to a final concentration of 1% for two hours in the dark, as described above. Briefly, plasma from COVID-19 patients and healthy controls were incubated with fluorescent-coded magnetic beads pre-coated with respective biotinylated peptides (Genscript) in a black 96-well clear-bottom plate for two hours at room temperature. After incubation, plates were washed five times with wash buffer (PBS with 0.05% PVA (Sigma-Aldrich) and 0.01% Tween (Promega)). Sample-antibody-bead complexes were incubated with PE-conjugated detection antibodies for one hour, washed five times with wash buffer and re-suspended in sheath fluid for acquisition on the FLEXMAP® 3D (Luminex) using xPONENT® 4.0 (Luminex) software. Data analysis was done on Bio-Plex Manager^TM^ 6.1.1 (Bio-Rad) and standard curves were generated with a 5-PL (5-parameter logistic) algorithm, reporting values for mean florescence intensity (MFI). The MFI values were logarithmically transformed to ensure normality. A cut-off value of mean + 3SD of logarithmically transformed MFI values of healthy controls was used as a baseline to classify the serological profile of COVID-19 patients as positive or negative.

***SARS-CoV-2 pseudovirus production***

The lentiviral based pseudovirus were produced as previously described.^2^ Briefly, using the third-generation lentivirus system, pseudotyped viral particles expressing SARS-CoV-2 Spike proteins of wildtype strain or other variants were generated by reverse transfection of 3 x 10^7^ of HEK293T cells (ATCC #CRL-3216) with 12 µg pMDLg/PRRE (a gift from Didier Trono, Addgene #12251), 6 µg pRSV-Rev (a gift from Didier Trono, Addgene #12253), 24 μg pHIV-Luc-ZsGreen (a gift from Bryan Welm, Addgene #39196) and 12 µg pTT5LnX-CoV-SP carrying Spike sequences of either WT Wuhan strain or each of the variant strains using Lipofectamine 2000 transfection (Invitrogen, #11668-019) according to the manufacturer’s instructions. After three days of culture, the viral supernatant was harvested by centrifugation to remove cell debris and filtered through a 0.45 µm filter unit (Sartorius). The viral titers were determined by Lenti-X^TM^ p24 Rapid Titre Kit (Takara Bio, #632500).

***Pseudovirus neutralization assay***

The pseudovirus neutralization assay was performed as previously described^2^ with slight modifications. CHO-ACE2 cells expressing human ACE2 protein on the surface^3^ (a kind gift from Professor Yee-Joo Tan, Department of Microbiology, NUS & IMCB, A*STAR, Singapore) were seeded at 1.8 x 10^4^ per well in a 96-well black polystyrene TC-treated microplate (Corning, #3904) in culture medium without Geneticin. After overnight culture, a single dilution (1:20) or serially diluted heat-inactivated plasma samples (from 1:10 to 1:6250) were incubated with equal volume of pseudovirus expressing SARS-CoV-2 Spike proteins of either the original wildtype or different variant strains (6 ng of p24) at a final volume of 50 µl at 37˚C for 1 hour, before being added to pre-seeded CHO-ACE2 cells in duplicate. After one hour of pseudovirus infection at 37°C, 150 µl of culture media was added to the pseudovirus infected cells and cells were cultured for additional two days. After wash with PBS, the cells were lysed with 1x Passive Lysis Buffer (Promega, #E1941) with gentle shaking at 125 rpm for 30 min at 37˚C and the luciferase activity was assessed with Luciferase Assay System (Promega, #E1510) on a GloMax Luminometer (Promega). The percent neutralization was calculated by normalizing the raw RLU values to the average value of RLU in virus only control wells, with calculated negative values plotted as zero.

***GenScript cPass Neutralization Antibody Detection kit***

Plasma samples were analyzed by the GenScript cPass Neutralization Antibody Detection kit, according to the manufacturer’s instructions. Briefly, the plasma samples were first diluted 1:10 in provided sample dilution buffer and then mixed with HRP-conjugated RBD with a volume ratio of 1:1 and incubated at 37°C for 30 min. The mixture was added to wells in the capture plate for another incubation at 37°C for 15 min. After washing, TMB solution was added to the wells and the plate was incubated in the dark for 15 min at 25°C. Absorbance at 450 nm was read immediately with Sunrise Microplate Reader (Tecan) after the addition of the stop solution.

***Roche Elecsys Anti-SARS-CoV-2 S and N immunoassay***

Plasma samples were analyzed by the Elecsys Anti-SARS-CoV-2 S (Elecsys-S) and Elecsys Anti-SARS-CoV-2 N (Elecsys-N) (Roche Diagnostics, Mannheim, Germany) immunoassays according to the manufacturer’s instructions, on cobas e411 analyzer. Briefly, plasma samples were first incubated with biotinylated SARS‐CoV‐2 S‐RBD‐specific (Elecsys-S) / biotinylated SARS‐CoV‐2 N-specific (Elecsys-N) recombinant antigen and SARS‐CoV‐2 S‐RBD‐specific (Elecsys-S) / SARS‐CoV‐2 N-specific (Elecsys-N) recombinant antigen labelled with a ruthenium complex, followed by another round of incubation with streptavidin-coated microparticles. The reaction mixture was then aspirated into the measuring cell where the microparticles were magnetically captured onto the surface of the electrode. Unbound substances were then removed with ProCell/ProCell M. A voltage was applied to the electrode to induce chemiluminescent emission, which was then measured by a photomultiplier. For the Roche S assay, the electro-chemiluminescent signal representing the level of antibodies was measured and samples within the linear range of quantitation (0.4 – 250 U/mL) were assigned a value. Samples with antibody levels ≥ 0.8 U/mL were considered positive. For the Roche N assay, the cut-off index (COI) was derived from the measured signal, where samples with COI ≥ 1.0 were considered reactive.

***Statistical analysis***

Statistical analysis was done using GraphPad Prism version 7.03 (GraphPad Software). EC_50_ values were calculated using Quest Graph™ EC50 Calculator from AAT Bioquest, Inc (https://www.aatbio.com/tools/ec50-calculator) with top and bottom constraints set at 100% and 0% respectively. To compare between multiple groups, Kruskal-Wallis tests and post hoc tests using Dunn’s multiple comparison tests were used to identify significant differences. For paired analysis between different variants, Wilcoxon matched-pairs signed rank tests or Friedman test followed by post hoc Dunn’s multiple comparisons tests were used. Pearson’s correlation analyses were performed to calculate correlation coefficient *r* value. *P* values less than 0.05 are considered significant.

***Data availability***

The data generated and analyzed during the current study are available from the corresponding author upon reasonable request.

***Role of the funding source***

This work was undertaken at Singapore Immunology Network (A*STAR), A*STAR Infectious Diseases Labs, and Bioinformatics Institute (A*STAR). This work was supported by the Biomedical Research Council (BMRC), the A*ccelerate GAP-funded project (ACCL/19-GAP064-R20H-H) (CIW and LR) from A*STAR, A*Cruse-funded projects (CIW, LR, SMS, LFPN) and NMRC COVID-19 Research fund COVID-19RF-001 (LFPN and LR), COVID-19RF-007 (LR, LFPN and CIW), COVID-19RF-060 (LFPN and LR), COVID-19RF-004 (SMS), and COVID-19RF-0008 (BEY, SP, LFPN, LR and CIW). GC is funded by the Singapore National Medical Research Council (NMRC) Open Fund Young Investigator Research grant (OFYIRG19nov-0051). This work was also supported by A*STAR, ARES Central Retained Earnings Held-in-Trust (LR).

***Author contributions***

Conceptualization: BW, YSG, SWF, BEY, JMC, SP, RTPL, DCL, SMS, LFPN, LR and CIW; Investigation: BW, YSG, SWF, EZXN, JMC, SNMS, NKWY, SNA, PXH, CYL, YHC, ZWC, MZT, AR, ATR, GC, MKS, RTCL, and YX; Supervision: SP, RTPL, YSL, DCL, SMS, LFPN, LR and CIW; Writing-Original draft: BW, YSG, and SWF; Writing-Review and editing: BW, YSG, SWF, BEY, JMC, RTPL, DCL, SMS, LFPN, LR and CIW.

***Declaration of competing interest***

A patent application for the SFB assay has been filed (Singapore patent 10202009679P: A Method Of Detecting Antibodies And Related Products. YSG, LFPN, and LR). A patent application on the identified linear epitopes S14P5 and S20P2 has also been filed (PCT/SG2021/050178: Antibody-binding linear B cell epitopes of SARS-CoV and SARS-CoV-2. NKWY, SNA, GC, and LFPN). All other authors declare no conflict of interest.

***ACKNOWLEDGMENTS***

We would like to thank all study participants who donated their blood samples for this study. We thank Professor Yee-Joo Tan (Department of Microbiology, NUS; Institute of Molecular and Cell Biology, A*STAR) who kindly provided CHO-ACE2 cells. We thank Dr Brendon John Hanson from DSO laboratories for pTT5LnX-CoV-SP plasmid. We are also grateful to the staffs from Singapore Immunology Network (SIgN) Multiplex Analysis of Proteins (MAP) platform, for their assistance in running multiplex microbead-based immunoassay.

Supplementary References

1. Goh YS, Chavatte JM, Lim Jieling A, et al. Sensitive detection of total anti-Spike antibodies and isotype switching in asymptomatic and symptomatic individuals with COVID-19. Cell Rep Med 2021; 2(2): 100193.
2. Poh CM, Carissimo G, Wang B, et al. Two linear epitopes on the SARS-CoV-2 spike protein that elicit neutralising antibodies in COVID-19 patients. Nat Commun 2020; 11(1): 2806.
3. Lip KM, Shen S, Yang X, et al. Monoclonal antibodies targeting the HR2 domain and the region immediately upstream of the HR2 of the S protein neutralize in vitro infection of severe acute respiratory syndrome coronavirus. J Virol 2006; 80(2): 941-50.
